# Mitochondria- and Oxidative Stress-Targeting Substances in Cognitive Decline-Related Disorders: From Molecular Mechanisms to Clinical Evidence

**DOI:** 10.1155/2019/9695412

**Published:** 2019-05-12

**Authors:** Imane Lejri, Anastasia Agapouda, Amandine Grimm, Anne Eckert

**Affiliations:** ^1^University of Basel, Transfaculty Research Platform Molecular and Cognitive Neuroscience, Basel, Switzerland; ^2^Neurobiology Lab for Brain Aging and Mental Health, Psychiatric University Clinics, Basel, Switzerland

## Abstract

Alzheimer's disease (AD) is the most common form of dementia affecting people mainly in their sixth decade of life and at a higher age. It is an extensively studied neurodegenerative disorder yet incurable to date. While its main postmortem brain hallmarks are the presence of amyloid-*β* plaques and hyperphosphorylated tau tangles, the onset of the disease seems to be largely correlated to mitochondrial dysfunction, an early event in the disease pathogenesis. AD is characterized by flawed energy metabolism in the brain and excessive oxidative stress, processes that involve less adenosine triphosphate (ATP) and more reactive oxygen species (ROS) production respectively. Mitochondria are at the center of both these processes as they are responsible for energy and ROS generation through mainly oxidative phosphorylation. Standardized *Ginkgo biloba* extract (GBE), resveratrol, and phytoestrogens as well as the neurosteroid allopregnanolone have shown not only some mitochondria-modulating properties but also significant antioxidant potential in *in vitro* and *in vivo* studies. According to our review of the literature, GBE, resveratrol, allopregnanolone, and phytoestrogens showed promising effects on mitochondria in a descending evidence order and, notably, this order pattern is in line with the existing clinical evidence level for each entity. In this review, the effects of these four entities are discussed with special focus on their mitochondria-modulating effects and their mitochondria-improving and antioxidant properties across the spectrum of cognitive decline-related disorders. Evidence from preclinical and clinical studies on their mechanisms of action are summarized and highlighted.

## 1. Introduction

### 1.1. Alzheimer's Disease: A Well-Known yet Untreatable Age-Related Neurodegenerative Disorder

Alzheimer's disease (AD), the most common neurodegenerative disorder, as well as dementia type, is characterized by extracellular senile beta-amyloid protein (A*β*) plaques and intracellular neurofibrillary tau tangles [1]. There are two types of AD: (i) the sporadic form of AD (SAD) whose onset occurs above the age of 65 and (ii) the familial AD forms (FAD) that are more rare with less than 1% occurrence among the AD cases and whose onset starts at a younger age (<65 years). The biological system of aging is the major risk factor of SAD [2]. The familial forms (FAD) bear inheritable mutations in the amyloid precursor protein (APP) and presenilin 1 and presenilin 2 genes [3, 4]. The symptoms of AD are the same in SAD and FAD [5]. There are different types of age-related cognitive diseases which differ in severity. SMI (subjective memory impairment) is the condition when nondemented aged people express subjective complaints related to their memory but have no organic or identifiable condition [6]. SMI is discussed as an early predictor of dementia [7–10]. The concept of mild cognitive impairment (MCI) defines an intermediate stage between normal aging and dementia. MCI patients show mild but measurable changes in cognitive tests and thinking abilities that are noticeable to the patients and to family members, but they are able to carry out everyday activities. Approximately 15-20% of people aged 65 or older have MCI. This group of people represents a population at increased risk for developing dementia, especially MCI involving memory problems [11]. The occurrence of MCI in the population is 3.2%, of which 11.1% of the cases convert to dementia within 3 years [12]. It has been indicated, yet not conclusively, that SMI is a precursor of MCI which can then lead to dementia or AD [4, 13]. Dementia is a more severe condition compared to SMI and MCI which affects aged people and interferes negatively in the performance of everyday activities. It is described as a cluster of symptoms related to mental, cognitive, and memory decline [12, 14]. There are different forms of dementia, such as AD, the most common type, and vascular dementia. Vascular dementia (VaD) is the second most common form of dementia and occurs as a cognitive decline due to a reduced blood flow in the brain (e.g., due to brain injury or stroke). However, sometimes different kinds of dementias coexist and their discrimination is difficult due to overlapping clinical symptoms. Moreover, many of these patients also suffer from psychiatric or behavioural problems that are sometimes referred to as BPSD (behavioural and psychological symptoms of dementia) or NPS (neuropsychiatric symptoms), including irritability, anxiety, psychosis, and aggression [15].

### 1.2. Mitochondria and Neuroplasticity

Mitochondria are essential yet independent organelles contained in eukaryotic cells, and they are responsible for numerous functional activities within the cells. However, they are not always an intrinsic structure of eukaryotic cells. They occur through the endosymbiosis of an alpha-proteobacterium into a prokaryotic progenitor, and this is why they contain their own DNA, namely, the mitochondrial DNA (mtDNA) [16]. Regarding the structural characteristics of these organelles, they contain two structurally and functionally distinct membranes, the outer and the inner membranes. The inner membrane encapsulates the matrix and also carries the electron transport chain (ETC) where oxidative phosphorylation (OXPHOS) is taking place. mtDNA is located in the matrix encoding 13 proteins which are used as structural components of the ETC complexes [17].

Mitochondria have obtained the title of “powerhouse of the cell” due to their ability of producing the energy, mainly through OXPHOS, required for the survival and functioning of the cell. Actually, they are more than just a “powerhouse” as they are the ultimate multitaskers which define the cell fate. Apart from the production of energy in the form of ATP, mitochondria are the key modulators of brain cell survival and death by controlling calcium (Ca^2+^) and redox equilibrium (which in turn affects neurotransmitter release and neuronal plasticity), by producing reactive oxygen species (ROS), and by controlling cell apoptosis [17–19]. The brain is an organ which requires a considerable amount of energy in order to operate, maintain, and enhance neuronal functions and plasticity. Neurons are postmitotic polarized cells with significant energy demands. OXPHOS, taking place in mitochondria, is the main energy provider in the form of ATP, and neurons depend almost solely on this procedure in order to satisfy their energy needs [20]. In particular, neurons direct this energy into the formation of interconnections, the synapses. The number and strength of these neuron interconnections define synaptic plasticity, which is responsible for cognitive function [21]. Synaptic plasticity is a crucial mechanism by which the neural activity generated by an experience alters synaptic transmission and therefore modifies brain function [22]. Neurite outgrowth is a process wherein developing neurons generate new projections as they grow in response to guidance cues. Nerve growth factors (NGF), or neurotrophins, are one family of such stimuli that regulate neurite growth [23]. Brain-derived neurotrophic factor (BDNF) exerts several actions on neurons ranging from the acute enhancement of transmission to long-term promotion of neurite outgrowth and synaptogenesis [24, 25]. Synaptic plasticity includes the dynamic regulation of long-term potentiation (LTP), spine density, and the number and length of dendrites and axons (neuritogenesis), as well as neurogenesis. Adult neurogenesis generates functional neurons from adult neural precursors in restricted brain regions throughout life [26]. These plasticity processes need a high energy requirement, and this is why mitochondria play such a pivotal role in the well-being of neurons especially when neurons need to adapt to periods of pathologically reduced functions.

### 1.3. Mitochondria, Oxidative Stress, Aging, and AD

However, while mitochondria regulate the functions of healthy neurons, they are also largely affected during aging and pathological states such as age-related neurodegenerative diseases. Mitochondria are not only the regulators of energy metabolism but are also the main ROS generators. ROS are immensely reactive species which are produced in mitochondria mainly by complexes I and III of the ETC when there is a leak of electrons. They are chemical species including hydroxyl radical (^∙^OH), superoxide anion (O_2_-), and hydrogen peroxide (H_2_O_2_) which can interact with and damage DNA and proteins and lipids which can compromise cell survival leading to aging and to vulnerability to several diseases [27, 28]. When they exist at normal levels, they constitute signalling agents in many physiological processes, such as redox homeostasis, cellular death, cellular senescence, and cell proliferation, and they can also trigger immune responses, synaptic plasticity, and cognitive enhancement [20, 27]. ROS are neutralized by antioxidant enzymes such as superoxide dismutase, which transforms the radicals (O_2_-) into H_2_O_2_, and by catalase, glutathione peroxidase, and thioredoxin peroxidase, which diffuse H_2_O_2_ [27]. In a healthy state, there is a balance between ROS production and neutralization. Nevertheless, when ROS are produced in excess, e.g., during aging, they directly affect mitochondria since mitochondrial membranes consist of long polyunsaturated fatty acids which are easily oxidized. Also, mtDNA is found in close proximity to the ROS source and is susceptible to mutations resulting in the production of faulty ETC proteins, leading back to the production of more ROS [18, 28]. It could be said that mitochondria are the main organelles in aging and neurodegeneration by being both generators and targets of ROS. It has been shown that aging is characterized by a rise in oxidative stress, a decline in antioxidant defense systems, and an impairment of the OXPHOS. So, aging is characterized by energy deprivation and a shift of the redox state towards oxidation. Mitochondria are at the center of these hallmarks [20]. Neurons, which highly depend on OXPHOS to satisfy their energy demands, are particularly susceptible to energy hypometabolism [20]. In addition, taking into account that they are nondividing cells, neurons are almost as old as the entire organism and are not replaced during life with the exception of the hippocampus that continuously generates new neurons during adulthood [20, 29]. This means that neurons accumulate oxidative stress and therefore defective mitochondria during aging [20, 30]. This is particularly important since mitochondrial dysfunction represents an early event in AD pathogenesis [20, 28, 31].

Intense oxidative stress and decreased brain energy metabolism are common characteristics of both normal aging and AD, although to different extents [20]. Of note, mitochondrial abnormalities are observed in FAD and SAD brains [32, 33]. On one hand, recent data obtained from AD models, in which mitochondrial failure is a prominent feature, implicate tau hyperphosphorylation as well as A*β* overproduction and deposition. On the other hand, A*β* and tau target mitochondria synergistically, thereby possibly amplifying each other's toxic effects. This interrelationship of A*β*, tau, and mitochondrial function constitutes a vicious cycle [34]. The mitochondrial cascade hypothesis postulates that mitochondrial dysfunction represents the most upstream pathology in AD [28]. According to this hypothesis, arresting brain aging will prevent the development of AD [32].

### 1.4. Mitochondria-Directed Natural Compounds

The current mitochondrial cascade hypothesis postulates mitochondrial dysfunction as a central pathomechanism in age-related degenerative disorders [28, 35, 36]. Taking into account their primary role in aging and in the early stages of AD, mitochondria constitute promising targets for therapeutic strategies. For this reason, pharmacological studies are directed in enhancing mitochondrial functions such as ATP production and respiration or in reducing mitochondrial harmful by-products such as ROS [36]. To date no drugs are able to cure or stop the progression of age-related neurodegenerative disorders. Most of them may be beneficial in delaying the progression of AD and only partially treat some of its symptoms (e.g., confusion and memory loss).

**Figure 1:**
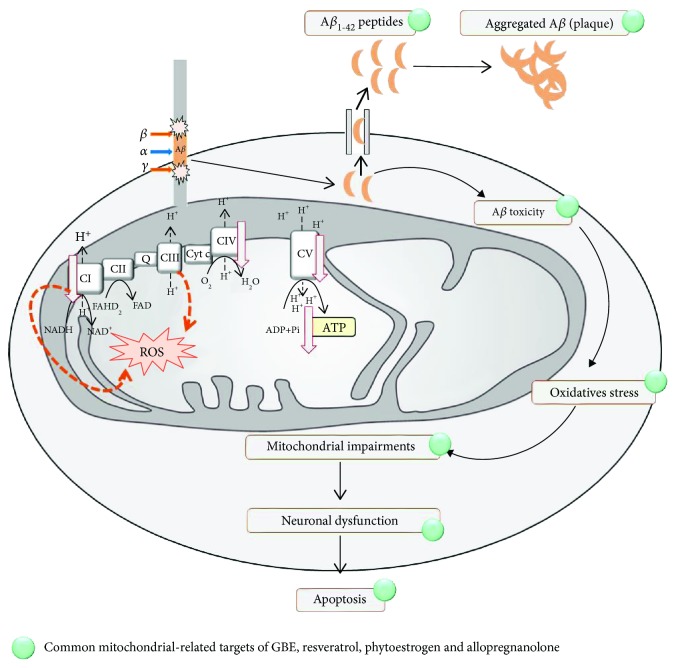
Common mitochondria-related targets of natural substances in neuroprotection. In AD, the precursor of amyloid protein APP is cleaved sequentially by *β*- and *γ*-secretases leading to the production of A*β* peptides, their aggregation, and the formation of extracellular plaques. Different A*β* species exist, but A*β*_1-42_ is one of the most abundant and is the one that is mainly deposited in the brain due to its hydrophobic and fibrillogenic nature. AD is associated with electron transport chain (ETC) impairments leading to decreased ATP levels and basal respiration, with a decrease of antioxidant defenses and an increase of ROS production by complex I and complex III (orange dashed arrows). Globally, *Gingko biloba*, resveratrol, and phytoestrogens have been shown to protect against cell death in AD through a common mechanism of action by reducing abnormal aggregation of A*β*, amyloid beta (A*β*) toxicity, oxidative stress, mitochondrial impairments leading to neuronal dysfunction, and apoptosis. *Gingko biloba*, resveratrol, and phytoestrogens are suggested to exert a beneficial effect in AD affected neurons, but their specific mechanisms of mitochondrial interaction are not fully described yet. ↓: AD-related decrease. The green circle indicates the common mitochondria-related targets of GBE, resveratrol, phytoestrogen, and allopregnanolone.

**Figure 2:**
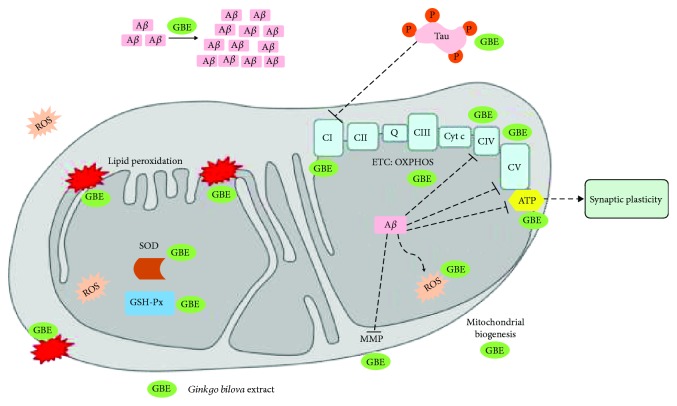
The effects of A*β*, hyperphosphorylated tau, and standardized *Ginkgo biloba* extract (GBE) on mitochondrial function in AD. It has been shown that mitochondrial dysfunction is a key feature in AD and plays a pivotal role on the onset of the disease. While defining the chronologically first hallmark of the disease can be puzzling, there is evidence about mitochondrial dysfunction being the first hallmark at the early stages of AD with A*β* occurring as a result. A*β* has been shown to cause a decline in OXPHOS, taking place at the ETC, which leads to defective complexes IV and V and decreased ATP production. Faulty OXPHOS function results in the production of ROS which, when in excess, cannot be counterbalanced by the antioxidant enzymes like GSH-Px and SOD. ROS can cause membrane lipid peroxidation and instable MMP. Hyperphosphorylated tau inhibits complex I activity. However, GBE has been proven to reduce A*β* aggregation and tau hyperphosphorylation and to enhance OXPHOS, activities of complexes, and ATP levels, as well as to restore MMP. ROS and consequently lipid peroxidation are reduced due to GBE, while the extract has the ability to enhance SOD and GSH-Px activity and also induce mitochondrial biogenesis. ↓: represents increase; ⟂: represents inhibition.

**Figure 3:**
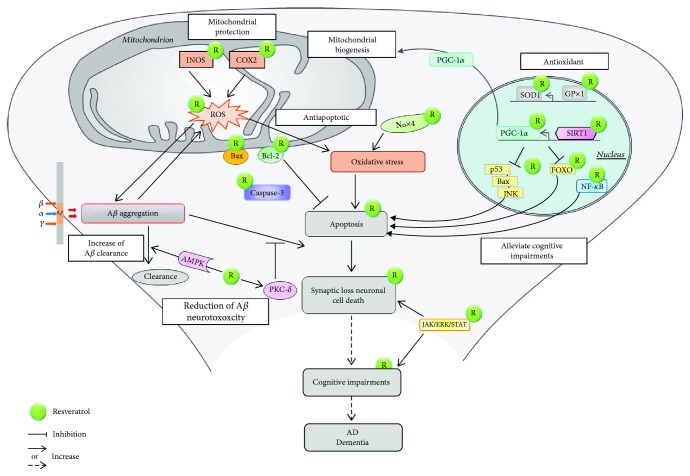
Neuroprotective effects of resveratrol in AD. The precursor of amyloid protein APP is cleaved sequentially by *β*- and *γ*-secretases leading to the production of A*β* and their aggregation. Resveratrol increases the clearance of A*β* peptides through the activation of AMPK. Resveratrol plays an important role in the neuroprotective properties as it reduces A*β* neurotoxicity by phosphorylating PKC-*δ*. Damaged mitochondria generate ROS which are implicated in apoptosis. iNOS and COX-2 also enhance the production of ROS. Resveratrol exerts antioxidant properties and attenuates oxidative damage by decreasing iNOS and COX-2 levels. Resveratrol also protects mitochondria by increasing the expression of ROS-inactivating enzymes GPx1 as well as SOD1 and by reducing the expression of the ROS-producing enzyme Nox4. Resveratrol also influences the A*β*-induced apoptotic signalling pathway by inhibiting the expression of caspace-3, Bax, FOXO, and p53 by blocking the activation of JNK and by restoring the decrease of Bcl-2 expression, as well as by inhibiting the increase of NF-*κ*B DNA binding. Mitochondrial biogenesis is induced by resveratrol through SIRT1 activation and deacetylation of PGC-1*α*. Resveratrol was also able to protect hippocampal neurons by alleviating cognitive impairment and reducing neuronal loss via modulating the janus kinases, extracellular signal-regulated kinases, and signal transducers, as well as the signalling pathway of the activators of transcription (JAK/ERK/STAT).

Many drugs including whole plant extracts and single compounds originate from natural and botanical sources. Two single compound AD drugs are derived from plants: (i) the acetylcholine-esterase inhibitor, galanthamine, derived from the *Galanthus* species (*Galanthus caucasicus* and *Galanthus woronowii*) and (ii) rivastigmine, a physostigmine analogue (physistigmine was isolated from the Calabar bean, *Physostigma venenosum*) [37, 38]. In addition, the phytopharmacon GBE that is used as antidementia medicine was shown to improve mitochondrial function emphasizing the concept of targeting mitochondria as an emerging and promising therapeutic approach [35, 39]. Therefore, we focused our search on natural compounds that possess mitochondria-enhancing properties based on our own past and ongoing research as well as on research of other groups. Standardized *Gingko biloba* extract (GBE), resveratrol, phytoestrogens, and the natural neurosteroid allopregnanolone fulfilled our criteria. Common targets of these agents (Figure 1) have been reported, such as ROS, mitochondrial membrane potential (MMP), A*β*, tau protein, antiapoptotic protein (Bcl-2), and OXPHOS (Figures 2 and 3). Accordingly, in this review we aimed to summarize the molecular modes of action of these natural agents with special focus on mitochondria, their mitochondrial function-enhancing properties, and their antioxidant properties. We discuss evidence on their mechanism of action from preclinical as well as clinical studies. Especially regarding clinical trials, there is a different level of existing evidence for each phytochemical. GBE, resveratrol, phytoestrogens, and allopregnanolone appear in a descending order according to their clinical evidence level. The databases PubMed and Google Scholar, as well as the database ClinicalTrials.gov were used for our search with a focus on the years 2000–2018. For the clinical evidence, we considered randomized, double-blind, placebo-controlled trials as well as ongoing trials, systematic reviews, meta-analyses, and Cochrane analyses.

## 2. Pharmacologic Features of Natural Substances in Alzheimer's Disease

### 2.1. Gingko biloba

*Gingko biloba* has existed for over 250 million years and is a native from Japan, Korea, and China; however, it can be found worldwide. Traditional Chinese clinicians originally utilized GBE for a variety of applications [40]. There are several *Ginkgo biloba* extracts sold on the market, including standardized and nonstandardized extracts, which are also used in studies. The standardized extracts have to meet specific criteria regarding their manufacturing process, the quality of the plant material, and their composition, which is not the case with the nonstandardized extracts. Many products have been reported on the market which are not standardized and are even adulterated. These products not only reduce the efficacy of GBE, but they can be potentially harmful [41]. GBE contains two main groups of active constituents ensuring its medicinal effects: terpenes (including bilobalide and ginkgolides A, B, and C) and flavonoids (including meletin, isorhamnetin, and kaempferol). Both the United States Pharmacopoeia and the European Pharmacopoeia define as standardized only extracts that contain the active components of *Ginkgo* in a certain and defined content. In particular, the standardized extracts should contain 5-7% triterpene lactones, 22-27% flavonoids, and less than 5 ppm of ginkgolic acids, which are toxic ingredients of *Ginkgo*. [42]. Most toxicological, pharmacological, and clinical investigations have focused on the neuroprotective value of two main standardized extracts labeled EGb761 and LI 1370 [43–45]. The EGb761 extract consists of 24% flavone glycosides (mainly quercetin, kaempferol, and isorhamnetin) and 6% terpene lactones (2.8-3.4% ginkgolides A, B, and C and 2.6-3.2% bilobalide), while the extract LI 1370 is composed of 25% ginkgo flavone glycosides as well as 6% terpenoids. Several terpene lactones (ginkgolides and bilobalide) show substantial mitochondria-protecting properties, while the flavonoid fraction seems to play an important role in the free radical scavenging properties [46]. In the following parts, only the effects of standardized GBE will be discussed.

#### 2.1.1. Mechanisms of Action Based on Preclinical Evidence

*2.1.1.1. Direct Effects of GBE on Mitochondria*. Several findings demonstrate the mitochondria-modulating effect of GBE, mainly in cellular and animal models of AD. In particular, GBE has been shown to attenuate effectively mitochondrial dysfunction through several mechanisms of action, such as antioxidant effect and free radical scavenging properties, with all the evidence leading to this conclusion having been reviewed extensively [35, 47–49]. *In vitro*, GBE was shown to ameliorate mitochondrial function by improving MMP and ATP levels at a low concentration of 0.01 mg/ml in pheochromocytoma cells (PC12) cells [46]. In amyloid precursor protein- (APP-) transfected human neuroblastoma cells, an AD cellular model with increased A*β* generation, GBE improved respiration of mitochondria, stimulated mitochondrial biogenesis, and increased ATP production [50]. Mitochondria-related modes of action of GBE are summarized in Figure 2.

*2.1.1.2. Effects of GBE on Oxidative Stress, Aβ, and Tau Toxicity Related to Damage of Mitochondria*. A*β* plaque deposition is one of the main hallmarks of AD. The overexpression of both A*β* itself and its precursor protein, the amyloid precursor protein (APP), has been used to create cellular and animal models of AD. GBE has been shown to be effective in reducing both the deposition of A*β* and its toxicity. In detail, the prooxidant A*β*_25-35_ peptide treatment was shown to decrease complex I and IV activities and to increase the level of reactive oxygen/reactive nitrogen species (ROS/RNS) in SH-SY5SY cells [51]. Thus, pretreatment with GBE was able to reduce the A*β*-related increase in ROS/RNS levels as well as to ameliorate the complex I and IV activities [51]. GBE protected against A*β*_1-42_ oligomer-induced neurotoxicity and cell damage with an indirect effect on SH-SY5Y neuroblastoma cells by improving Hsp70 protein expression and subsequently by activating the Akt (protein kinase B) pathways as well as ER stress [52]. GBE also attenuated A*β*_1-42_ oligomer-induced cell damage and protected against A*β* toxicity and oxidative stress [53, 54], as well as apoptosis [52]. GBE was also able to reduce A*β* production [55]. In terms of animal models, a chronic treatment with GBE improved cognitive defects in a transgenic mouse model of AD (Tg2576), a model that overexpresses a mutant form of APP [53]. GBE was also shown to decrease A*β* oligomers and to significantly increase neuronal proliferation in the hippocampus of young (6 months) and old (22 months) mice in a double transgenic mouse model (TgAPP/PS1) [54]. A chronic daily treatment with GBE for 6 months improved the cognitive function and alleviated amyloid plaque deposition in two-month-old APP/PS1 mice. Of note, GBE treatment seems to decrease the level of insoluble A*β*, while the soluble content of A*β* was unchanged [56]. GBE reduced the hyperphosphorylation of tau at AD-specific Ser262, Ser404, Ser396, and Thr231 sites, rescued the activity of tau phosphatase PP2Ac and kinase GSK3*β*, and reduced the oxidative stress in the hippocampus and prefrontal cortex on a hyperhomocysteinemia-treated rat model of AD. Memory lesions were also restored, and the expression of synapse-associated protein PSD95 and synapsin-1 protein was upregulated [57].

*2.1.1.3. Effects of GBE on Neuroplasticity Pathways*. GBE exerts its beneficial effects not only by acting on the Akt pathway, as aforementioned, but also by acting on the cyclic AMP response element-binding protein (CREB) [54, 58, 59]. CREB phosphorylation induces transcriptional activation which results in the expression of BDNF, and therefore, in synaptic plasticity and cognitive enhancement. Conversely, lack of CREB phosphorylation is a pathological ailment of neurodegenerative diseases such as AD [60].

**Figure 4:**
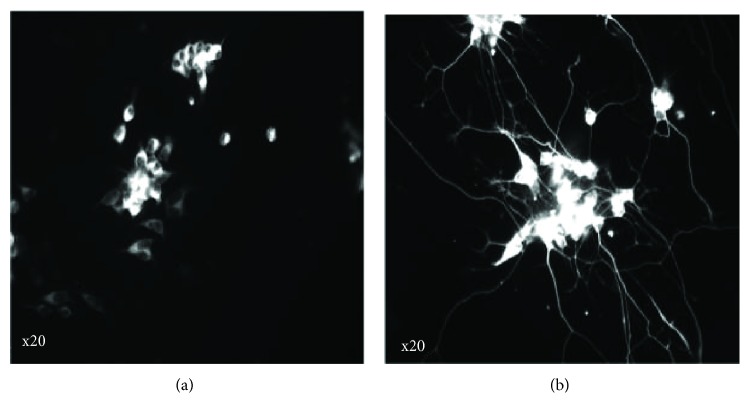
Standardized *Ginkgo biloba* extract (GBE) LI 1370 (Vifor SA, Switzerland) (100 *μ*g/ml) increased neurite outgrowth of SH-SY5Y neuroblastoma cells after 3 days of treatment in 3D cell culture. Pictures were taken using a cell imaging multimode reader Cytation3 (Biotek Instruments Inc., X20 in black and white) after immunostaining (IMS, *β*III-tubuline/Alexa488). Compared to the untreated SH-SY5Y cells (CTRL, (a)), 100 *μ*g/ml of GBE (b) was efficient in increasing the formation of neurites.

In detail, the administration of GBE restored CREB phosphorylation in the hippocampus of TgAPP/PS1 mice [54]. Quercetin and bilobalide are the major constituents that have contributed to GBE-induced neurogenesis [58]. Both constituents promoted dendritic processes in hippocampal neurons and restored A*β* oligomer-induced synaptic loss, as well as restored CREB phosphorylation [58]. Ginkgo flavonols quercetin and kaempferol have been shown to stimulate BDNF and phosphorylation of CREB in neurons isolated from double transgenic AD mouse (TgAPPswe/PS1e9) [59]. Recently, our team could confirm the neurite outgrowth stimulating effects of GBE in a 3D cell culture model (Figure 4).

#### 2.1.2. Clinical Evidence

Apart from the preclinical studies, the extract has been largely investigated in clinical trials in a range of age-associated cognitive conditions from SMI and MCI to dementia and AD. GBE has been suggested for both the symptomatic treatment and prevention of those cognitive decline-related diseases. The standardized GBE is considered a phytopharmacon, and the dose of 240 mg/day is recommended as the most effective by the guidelines for biological treatment of dementias [12]. There are 9 categories (A, B, C, C1, C2, C3, D, E, and F) and 5 grades (1-5) of pharmaceuticals used for AD and other dementias according to the level of existing clinical evidence and the occurrence of side effects, respectively. GBE belongs to category B of the level of evidence (limited positive evidence from controlled studies) and to grade 3 [12]. Here, we are going to highlight evidence on the extract's efficacy on subgroups of age-associated cognitive conditions in an ascending severity order (Table 1).

*2.1.2.1. Patients with SMI and MCI*. Three randomized, double-blind, placebo-controlled, parallel-group trials were conducted for patients with memory complaints, one in SMI and two in MCI patients. In total, data from 61 SMI and 460 MCI patients were evaluated. One trial conducted in healthy aged patients with SMI showed that GBE enhanced cognitive flexibility without changes in brain activation and that it mildly increased prefrontal dopamine [61]. Two trials showed that GBE ameliorated neuropsychiatric symptoms (NPS) and cognitive ability in patients with MCI [62] as well as improved cognitive functioning and aspects of quality of life in subjects with very mild cognitive impairment [63].

*2.1.2.2. Patients with Dementia*. GBE has been found particularly efficacious in demented people with neuropsychiatric symptoms (NPS) [64, 65]. In total, 3 original papers, 1 systemic review, 6 meta-analyses, and 1 Cochrane analysis involving 14974 demented patients were evaluated. In detail, the pooled analyses of 4 published trials in a systemic review, involving outpatients with mild to moderate dementia and BPSD, demonstrated the efficacy of GBE at a daily dose of 240 mg [66]. Six meta-analyses (3 trials included in these meta-analyses were conducted in 1997 [67–69]) of 32 controlled, randomized, double-bind clinical trials and one bivariate meta-analysis of 6 trials come to the conclusion that GBE is efficacious and well tolerated in patients with a diagnosis of AD, VaD, or mixed dementia in three typical domains of assessment in dementia, i.e., cognition, activities of daily living (ADL), and clinical global judgment [65, 70–74]. However, there are also the studies with inconclusive or contrasting results to the efficacious effect of GBE in demented subjects [75–77].

*2.1.2.3. Patients with Specific Dementia Type: AD and Vascular Dementia*. In total, data from 1 original paper, 1 review, and 3 systematic reviews and meta-analyses involving 6880 patients with AD and VaD were evaluated. In detail, in an original paper, low doses of GBE administered to patients with vascular cognitive impairment in a randomized, double-blind, placebo-controlled trial showed significant deceleration of cognitive decline versus placebo only in one (Clinical Global Impression) of the four tests conducted in the trial [78]. The systematic reviews and meta-analyses (3 trials included in these meta-analyses were conducted in 1997 [67–69]) concluded that GBE exerts potentially beneficial effects on the improvement of activities of daily living, cognitive function, and on global clinical assessment in patients with MCI or AD, in mainly the AD type of dementia and in aged people with VaD having NPS [79–82].

*2.1.2.4. Prevention*. The preventive effect of GBE was reported in 14812 patients in three original papers and one systematic review and meta-analysis. In contrast, there are 4 studies that do not support the efficacious effect of GBE in preventing the onset of AD in either healthy aged or aged with MCI people [83–86]. The outcome for the efficacy of GBE in preventing the onset of AD in healthy individuals varies among different studies. However, there is also high variability in the design of the studies in terms of GBE doses, duration of the treatment, sample size, statistical tools, and compliance with the medication. Therefore, there is space for criticism regarding the methodological design of studies and the interpretation of the outcome. There are two large studies which form good examples of scepticism towards their negative outcome: the GEM study and the GuidAge study [83, 84, 87]. The GEM study was conducted in healthy old people (80 years old or more) and found no efficacy of GBE in preventing the onset of AD. In this study, the compliance of subjects with the treatment was nonadequate, yet this parameter was not taken into account in the interpretation of the results. In the GuidAge study, the conversion rate from memory complaints to dementia was examined in aged people with memory complaints and no difference was found between GBE and placebo. However, the statistical power for the analysis of hazards was found low. The selection of suitable statistical methods to take into account increasing hazards overtime is crucial for meaningful results and increased significance [35].

Based on the included studies, GBE has been reported in only a few studies that show no effect. The majority of the recent studies demonstrated that the treatment with doses up to 240 mg/day was safe, well-tolerated, and efficacious against age-related disorders.

In summary, GBE has been proven more effective in patients with cognitive impairment at baseline than preventing the onset of cognitive impairment in healthy aged subjects. As mentioned before (see Introduction), mitochondrial dysfunction is more profound in cognitive disorders than in normal aging. Similarly, GBE shows increasing promising effects with increasing cognitive impairment. This, again, represents an indicator that GBE exerts its effects clinically by acting on mitochondria [35]. Thus, we can conclude that GBE can potentially improve mitochondrial dysfunction across the aging spectrum.

### 2.2. Resveratrol

Resveratrol, known as a polyphenol from white hellebore (*Veratrum grandiflorum*), was discovered by Takaoka (1939) as a component of several dietary sources such as berries, peanuts, and red grape skin or wine. Siemann and Creasy discovered that resveratrol is present at high concentration in red wine [88]. Resveratrol has been reported to possess several benefits, including antitumor, antioxidant, antiaging, anti-inflammatory, cardioprotective, and neuroprotective properties. This polyphenol has emerged as a novel natural agent in the prevention and possible therapy of AD [89].

#### 2.2.1. Mechanisms of Action Based on Preclinical Evidence

*In vitro* and *in vivo*, the direct molecular targets of resveratrol are not known in detail. However, there is evidence that resveratrol exerts a complex mode of actions through the protection of mitochondrial function and the activation of biogenesis, through its effect on certain signalling pathways, through its antioxidant effects, through the increase of A*β* clearance, and through the reduction of A*β* neurotoxicity [90] (Figure 3).

*2.2.1.1. Direct Effects of Resveratrol on Mitochondria*. Dietary supplementation with 0.2% (*w*/*w*) resveratrol suppressed the aging-associated decline in physical performance in senescence-accelerated mice (SAMP1) at 18 weeks of age by improving several mitochondrial functions such as the activity of respiratory enzymes, oxygen consumption, and mitochondrial biogenesis, as well as the activity of lipid-oxidizing enzymes [91]. In 18-month-old aged mice, resveratrol (15 mg/kg/day) and/or exercise for 4 weeks were able to counteract aging-associated oxidative damage targeting mitochondrial biogenesis and function by causing overexpression of peroxisome proliferator-activated receptor-gamma coactivator (PGC-1*α*) mRNA and by increasing citrate synthase enzyme activity [92]. Mitochondrial biogenesis is induced by resveratrol through SIRT1 activation and deacetylation of PCG-1*α* [90] (Figure 3).

*2.2.1.2. Effects of Resveratrol on Oxidative Stress*. Damaged mitochondria activate ROS production during oxidative stress which is involved in apoptosis [93]. ROS may damage the mitochondrial and cellular proteins and nucleic acids, causing lipid peroxidation and resulting in the loss of membrane integrity [94] (Figure 3). Resveratrol also protects mitochondria by increasing the expression of the ROS-inactivating enzymes glutathione peroxidase 1 (GPx1) and superoxide dismutase 1 (SOD1) and by reducing the expression of the ROS-producing enzyme NADPH oxidase 4 (Nox4) [93, 95] (Figure 3). In line with this, resveratrol rescued A*β*-treated human neural stem cells (hNSCs) from oxidative stress by increasing the mRNA of antioxidant enzyme genes such as SOD-1, nuclear factor erythroid 2-related factor 2 (NRF-2), Gpx1, catalase, and heme oxigenase 1 (HO-1) [96]. In addition, resveratrol exerted antioxidant properties and attenuated oxidative damage by decreasing iNOS and COX-2 levels [93].

*2.2.1.3. Effects of Resveratrol on Aβ Toxicity Related to Damage of Mitochondria*. Thanks to its natural antioxidant properties and/or by sirtuin1 (SIRT1) activation, resveratrol shows a neuroprotective effect because it counteracts A*β* toxicity. In more details, resveratrol increases the clearance of A*β* through the activation of AMPK [90] (Figure 3). This natural molecule plays an important role in reducing A*β* neurotoxicity by phosphorylating protein kinase C delta (PKC-*δ*) [90] (Figure 3). Resveratrol also influences the A*β*-induced apoptotic signalling pathway through SIRT1 activation, including inhibiting the expression of caspase protein 3 (caspase-3), apoptotic regulator Bax, Forkhead box O (FOXO), and tumor protein p53, through blocking the activation of c-Jun N-terminal kinase (JNK) and restoring the decrease of B-cell lymphoma 2 (Bcl-2) expression, as well as through inhibiting the increase of the nuclear factor kappa-light-chain-enhancer of activated B cell (NF-*κ*B) DNA binding [90] (Figure 3). Resveratrol (20 *μ*M) protected PC12 cells against neurotoxicity caused by A*β*_25-35_ by provoking autophagy which was proven dependent on the tyrosyl tRNA synthetase-poly(ADP-ribose) polymerase 1 (TyrRS-PARP1) and SIRT1 pathway (TyrRS-PARP1-SIRT1 pathway) [97]. A very low concentration of resveratrol (0.2 mg/l) significantly attenuated A*β* neuropathology and AD-type deterioration of spatial memory function in Tg2576 mice compared to control [98]. In a transgenic mouse model of AD (Tg19959), dietary supplementation with resveratrol (300 mg/kg) decreased amyloid plaque formation [93]. In order to translate the animal doses into ones that are relevant in humans, a scaling factor of 0.08 is used to calculate the human equivalent dose (http://www.fda.gov/cber/gdlns/dose.htm). For resveratrol, this is about 24 mg/kg or 1.68 g per day for a 70 kg individual [93]. Resveratrol is also known to act as a phytoestrogen (this mode of action of resveratrol is discussed in more detail in Phytoestrogens).

*2.2.1.4. Effects of Resveratrol on Metabolic and Signalling Pathways*. Resveratrol has been suggested to regulate cellular processes by activating key metabolic proteins such as SIRT1, 5′ adenosine monophosphate-activated protein kinase (AMPK), and peroxisome proliferator-activated receptor gamma coactivator 1-alpha (PCG-1*α*) [99–101]. Sirtuins and nicotinamide adenine dinucleotide- (NAD^+^-) dependent protein deacetylases are described as novel therapeutic agents against neurodegenerative disease pathogenesis [102]. In fact, the essential neuroprotective effect of resveratrol is based on the action of SIRT1 and AMPK and on the phosphorylation/acetylation status of PGC-1*α* that consequently activates the mitochondrial biogenesis leading to the improvement of the mitochondrial activity [103] (Figure 3).

In a study using A*β*-treated hNSCs, the neuroprotective effect of (10 *μ*M) resveratrol was demonstrated by the activation of the AMPK-dependent pathway by rescuing the expression levels of inhibitory kappa B kinase (IKK) and by restoring iNOS and COX-2 levels [104]. In the inducible p25 transgenic mouse model of tauopathy and AD, resveratrol-mediated (5 *μ*g/*μ*l) SIRT1 activation reduced learning impairment and hippocampal neurodegeneration [105]. The JAK/ERK/STAT signalling pathway (janus kinases, extracellular signal-regulated kinases, and signal transducers and activators of transcription) is implicated in cell survival, proliferation, and differentiation, while the dysregulation of the JAK/STAT pathway in neurodegenerative disorders contributes to neuronal loss, cognitive impairment, and brain damage [96]. Treatment with 20 mg/kg resveratrol exerted a neuroprotective effect via the JAK/ERK/STAT signalling pathway in a rat model of ischemia-reperfusion injury. In detail, resveratrol attenuated the increase in phosphorylation of JAK, ERK, STAT, and JNK caused by ischemia-reperfusion [96] (Figure 3).

#### 2.2.2. Clinical Evidence

Only eight clinical trials and four ongoing trials on resveratrol aim at evaluating the effects of this compound on cognitive function in humans [106] (Table 2). Efficacy results of resveratrol are based only on one clinical trial in MCI and one in AD patients.

*2.2.2.1. Young and Old Healthy Subjects*. Witte et al. conducted a study to evaluate the effect of resveratrol (200 mg/day) supplementation in a formulation with quercetin 320 mg in 23 healthy overweight older individuals versus placebo during 26 weeks. They showed that resveratrol supplementation is able to improve memory performances and glucose metabolism and is able to increase hippocampal functional connectivity in older adults for the maintenance of brain health during aging [107]. No effect on cognitive function was detected in young healthy people [94, 95].

*2.2.2.2. Patients with Cognitive Decline and MCI*. Lee et al. examined the effects of grape consumption (which contains resveratrol) on cognitive function and metabolism in the brain of patients with mild cognitive decline and demonstrated a protective effect of the grape extract against pathologic metabolic decline [108]. In a more recent 14-week study carried out on 80 postmenopausal women aged 45-85 years, it was proven that a regular consumption of a modest dose of resveratrol (75 mg twice daily) is able to enhance cerebrovascular function and cognition and to reduce their heightened risk of accelerated cognitive decline [109].

Clinical studies are underway to explore the beneficial effect of resveratrol on MCI. In the ongoing trials, one four-month resveratrol supplementation study in phase 1 aims at evaluating the efficacy and safety of bioactive dietary preparation (BDPP) at low, moderate, and high doses in treating mild cognitive impairment on 48 MCI subjects (55-85 years) [110]. The purpose of another study in phase 4 is to test the effect of a six-month administration of resveratrol on brain functions in MCI subjects (50-80 years) (National Institutes of Health, ClinicalTrials.gov) [111]. In a randomized, double-blind interventional study, resveratrol intake (200 mg/day, 26 weeks) reduced glycated hemoglobin A1c, preserved hippocampus volume, and improved hippocampus resting-state functional connectivity (RSFC) in 40 well-characterized patients with MCI (21 females, 50-80 years) [112].

*2.2.2.3. Patients with Moderate AD and Dementia*. Class II evidence provided by the study of Turner et al. on patients with AD showed that resveratrol (500 mg/day to 2 g/day, 52 weeks) is well-tolerated, safe, and able to decrease A*β*_40_ levels in cerebrospinal fluid (CSF) and plasma but had no significant effects on cognitive score [113]. Recently, a phase 2 study was conducted investigating the effect of resveratrol (500 mg) in individuals with mild to moderate AD confirming its tolerability and safety as well as its modulation of AD biomarker pathways [114]. Currently, an ongoing study in phase 3 tests the effect of resveratrol supplementation (215 mg/day for 52 weeks) on cognitive and global functioning in mild-to-moderate AD patients (50-90 years) [115]. A second ongoing study in phase 3 aims at evaluating the effect of resveratrol combined with glucose and malate in slowing down the progression of AD after 12 months in mild-to-moderate AD (50-90 year old patients) [116].

On the basis of the results from the very few clinical trials in MCI and AD, no conclusion about the efficacy of resveratrol on cognition can be drawn at the current time, but promising trials are underway.

### 2.3. Neurosteroids

Neurosteroids offer therapeutic opportunities through their pleiotropic effects on the nervous system. They are a subcategory of steroids synthetized de novo from cholesterol in the central nervous system independently of supply by peripheral steroidogenic glands [117, 118] and accumulate within the brain in neurons or glial cells [119, 120]. Neurosteroids are derived from cholesterol which is translocated from the outside to the inside of mitochondria via the translocator protein (TSPO). In the inner mitochondrial membrane, cholesterol is then converted by the cytochrome cholesterol side-chain cleavage enzyme (P450scc) to pregnenolone, the precursor of all the neurosteroids [121]. In particular, pregnenolone and allopregnanolone play an essential role in aging, in the performance of memory, and in physiopathology. Indeed, the age-related drop of neurosteroids gives rise to neuronal degeneration and dysfunction in human and animal models owing to the loss of neurosteroid neuroregenerative and protective effects [122, 123]. Allopregnanolone is used in several studies as a plasmatic biomarker for AD because of its reduced level in the plasma of demented patients [122]. It is known to be a regenerative agent in the brain [124]. Several neurosteroids were quantified and were found decreased in postmortem brains of aged nondemented controls and aged AD patients. The transgenic mice model of AD (APPswe+PSEN1Δ9 mice) presents a decreased ability to form allopregnanolone in the hippocampus [125].

#### 2.3.1. Allopregnanolone

*2.3.1.1. Mechanisms of Action Based on Preclinical Evidence*. *2.3.1.1.1. Direct Effects of Allopregnanolone on Mitochondria*. In control and APP/A*β* SH-SY5Y cells, allopregnanolone improved basal respiration and glycolysis as well as increased the bioenergetic activity and ATP production [126]. In APP-transfected cells, a pretreatment with allopregnanolone exerted a neuroprotective effect against oxidative stress-induced cell death via the amelioration of the cellular and mitochondrial energy, the reduction of ROS, and the improvement of mitochondrial respiration [126]. Thereby, it exerted its beneficial effect by improving the mitochondrial redox environment, such as MnSOD activity and mitochondrial ROS levels [127]. Moreover, allopregnanolone increased ATP levels and respiration in mouse primary cortical neurons [127]. In addition, *in vitro*, allopregnanolone potentiated mitochondrial respiration in both adult neural stem cells (NSCs), neurons, and mixed glia [128]. *In vivo*, allopregnanolone was able to restore the ovarectomized- (OVX-) induced decrease in mitochondrial respiration in both non-Tg and 3xTgAD mice [128]. Moreover, allopregnanolone also improved the activity of bioenergetic enzymes such as pyruvate dehydrogenase (PDH) and *α*-ketoglutarate dehydrogenase (*α*KGDH) [128].

**Figure 5:**
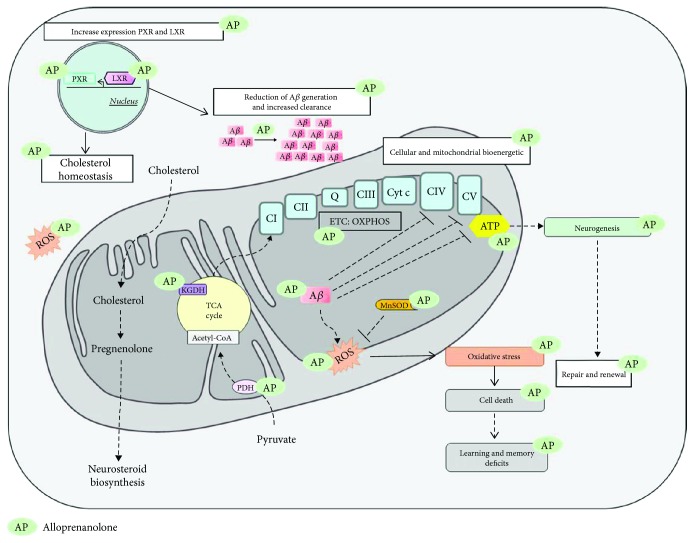
Neuroprotective effects of allopregnanolone (AP) in AD. AP has been proven to reduce A*β* aggregation-induced cell death. It exerts a neuroprotective effect against oxidative stress-induced cell death via the improvement of the cellular and mitochondrial energy by enhancing the OXPHOS and ATP levels. AP ameliorates the mitochondrial redox environment by decreasing ROS and by improving the activity of the enzyme MnSOD. AP also has beneficial effects on bioenergetic enzymes such as PDH and *α*KGDH implicated in the TCA cycle. AP ameliorates cholesterol homeostasis and clearance for the biosynthesis of neurosteroids by raising the expression of PXR and LXR. AP promotes repair and renewal of neurons leading to restored cognitive performances in AD.

*2.3.1.1.2. Effects of Allopregnanolone on Aβ Toxicity Related to Damage of Mitochondria*. In a recent study, allopregnanolone was shown to exert an increased neuroprotective activity against A*β*_42_-induced cell death in neural stem cells [129] (Figure 5). *In vivo*, the natural neurosteroid allopregnanolone appears to be a promising therapeutic tool for the development of neurogenic and/or neuroprotective strategies, but diverse points have to be taken into account, including the dosing regimen, the treatment regimen, bioavailability, solubility, route of administration, and sex differences. Acute single administration of allopregnanolone promoted neurogenesis in the subgranular zone (SGZ) in the triple transgenic mouse model of AD (3xTgAD) at 3 months of age prior to the appearance of AD [71]. Allopregnanolone reversed memory and learning deficits in these mice. Chen et al. showed that allopregnanolone administration (once/week for 6 months) decreased A*β* generation and promoted survival of newly generated neurons in the brain of 3xTgAD [130]. They also demonstrated that allopregnanolone increased oligodendrocyte myelin markers and ameliorated cholesterol homeostasis and clearance from the brain by increasing the expression of PXR and Liver-X-receptor (LXR). Singh et al. reported that allopregnanolone is able to restore cognitive performance in the preplaque phase of AD as well as memory and learning in aging 3xTgAD mice [131]. All these studies demonstrated the neuroprotective effects of allopregnanolone against the A*β* toxicity in 3xTgAD mice and also its capacity to stimulate rodent and human neural progenitor cell proliferation and to compensate the cell loss [130, 132]. Continuous infusions of allopregnenanolone were antiregenerative, while intermittent administration promoted repair and renewal in a mouse model of AD [124]. The mode of action of allopregnanolone is summarized in Figure 5.

*2.3.1.2. Clinical Evidence*. Currently, there is only one phase I ongoing clinical trial testing the safety and the tolerability of allopregnanolone in patients with mild cognitive impairment and early AD [133] (Table 3). The primary aim of this phase 1 study is to evaluate the maximally tolerated dose after intravenous injection of allopregnanolone (2, 4, or 6 mg, once per week for 12 weeks). Thus, no clinical evidence is currently available.

The natural neurosteroid allopregnanolone appears to be a promising therapeutic tool with specific regard to its neurogenic properties besides its mitochondria-directed effects. However, more trials are urgently needed to prove that.

### 2.4. Phytoestrogens

Phytoestrogens are the most bioactive molecules of soy and present structural similarity to the 17*β*-estradiol, which is the major circulating estrogen. Specific estrogen receptors have been shown to localize in mitochondria in the frontal lobe and the hippocampus of men and women suggesting a role of estrogen in controlling cognitive functions and memory processes via energy supply [134]. Estrogen plays a neuroprotective role during the aging process, especially through its beneficial impact upon mitochondrial metabolism by increasing glucose utilization by cells as well as by enhancing ETC activity, by stabilizing the MMP, and by preventing ROS production and calcium-induced excitotoxicity [135]. Moreover, females live longer than males and this can be attributed in part to the antioxidant effect of estrogen and the upregulation of life longevity-related genes [19, 136]. The phytoestrogens are characterized by their ability to bind to estrogen receptor *α* and estrogen receptor *β* and to exert similar responses to endogenous estrogens [137]. Isoflavones are a subclass of phytoestrogens and are contained abundantly in soy and soybeans. Soy presents estrogenic effects attributed to genistein, daidzein, and glycitein. The most potent isoflavone is genistein, while daidzein and glycitein present an affinity to the estrogen receptor, 100-500 times lower than genistein [138]. Estrogen receptors are localized in the important brain areas, including the prefrontal cortex and the hippocampus that are also known to be vulnerable to age-related decline [139–142].

#### 2.4.1. Mechanisms of Action Based on Preclinical Evidence

*2.4.1.1. Effects of Phytoestrogens on Aβ and Tau Toxicity and Cognitive Performance Related to Damage of Mitochondria*. One of the most important phytoestrogens is resveratrol, an estrogen receptor agonist/antagonist. In particular, resveratrol acts on estrogen receptor *β*, whose activation is known to play a major role in cognitive processes, leading to the improvement of cognitive impairment in AD [143]. The soybean is a source of vegetable proteins and contains also other functional ingredients including phytoestrogens. The isoflavones genistein and daidzein have been shown to present protective effects against tau protein phosphorylation [144]. Animal models confirmed the neuroprotective effects of phytoestrogens. Genistein, the most active molecule of soy isoflavones, improved A*β*-induced cell death and reduced neuronal loss in rats [145–147]. In OVX female rats, dietary supplementation of soy phytoestrogens (0.4 g/kg or 1.6 g/kg) or 17*β*-estradiol (0.15 g/kg) for 12 weeks has been shown to increase the expression of brain neurotrophic factors such as BDNF and tropomyosin receptor kinase B (TrkB) and, as a result, to ameliorate hippocampal learning [148]. In normal and OVX transgenic AD mice, a selection of phytoestrogens in combination, composed of genistein, daidzein, and equol, has been shown to improve spatial working memory performance and to reduce mortality, as well as to delay neuropathological changes associated with AD [149].

**Figure 6:**
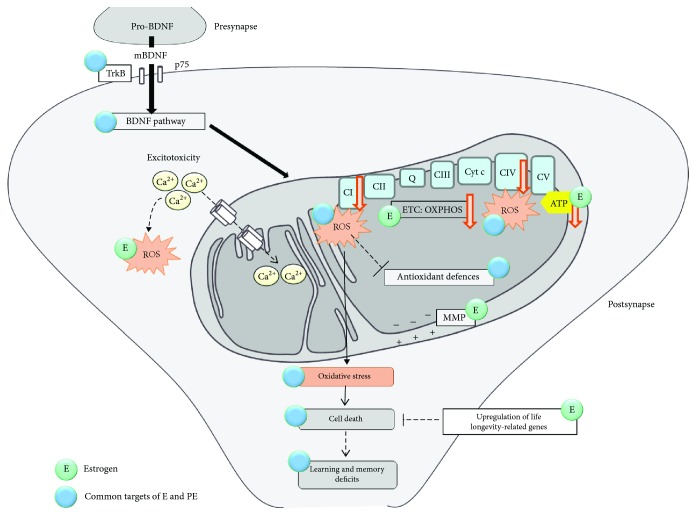
Modulation of mitochondrial function by estrogen and phytoestrogen. Less evidence is provided for the direct effects of phytoestrogen on mitochondria compared to estrogen, but antioxidant properties were demonstrated.

*2.4.1.2. Effects of Phytoestrogens on Oxidative Stress*. The phytoestrogens are also known for their neuroprotective antioxidant effects in neuronal cell models after exposure to neurotoxic substances [150–152]. Phytoestrogens are able to reduce ROS within a cell and to protect from cellular damage [153]. In aged mice, soybean supplementation has been shown to prevent cognitive deficits by decreasing free radical generation, by enhancing scavenging of free radicals, and by increasing GSH levels [154]. Compared to estrogen itself, less evidence is provided for the direct effects of phytoestrogens on mitochondria, but antioxidant properties were demonstrated [155–158]. The molecular effects of phytoestrogens are summarized in Figure 6.

#### 2.4.2. Clinical Evidence

Until today, no clinical trials in MCI and AD were performed. Thus, currently there is no clinical evidence.

*2.4.2.1. Healthy and Postmenopausal Women*. Among five randomized controlled trials, four recent studies reported the beneficial effect of phytoestrogens on cognitive function in healthy individuals (Table 4). In a study with young healthy adults of both sexes, a high soya or a low soya diet for 10 weeks had a beneficial effect and showed significant improvements in short-term and long-term memory as well as in mental flexibility [159]. In another cross-over design study, the administration of 4 capsules/day containing soya isoflavones during 6 weeks improved the spatial working memory of men aged 30-80 years [160]. In postmenopausal women, 6 months of treatment duration with isoflavone supplementation provoked better learning, mental flexibility, and increased attention, as well as caused improvement in mood and lower depressive symptoms [161]. In a small mixed gender sample of older adults, soy supplementation ameliorated the visuospatial memory and the construction of verbal fluency and speeded dexterity [162]. All these studies demonstrated that phytoestrogens may affect human cognition. However, no clinical trials of phytoestrogens are known for the prevention or the treatment of AD.

Inconclusive findings have also been reported from randomized controlled trials and observational studies in humans. In fact, these discrepant data could have several possible reasons. Investigation in European cohorts showed that a low dietary consumption of phytoestrogens had a significant effect on the improvement of the quality of life but no effect on cognition [163].

Mediating variables in the characteristics of the study population such as gender, age, ethnicity, and menopausal status appears to play an important role [164]. Phytoestrogens have been shown to have time-limited positive effects on cognition. These findings are in line with estrogen treatment which also exerts an initially positive short-term effect on cognition and a reversion after a long-term continuous use in aged women [164].

Globally, the effects of phytoestrogens can be dependent upon a window of opportunity for treatment and can affect males differentially than females due to the diminished presence of ER-mediated protective mechanisms and the tyrosine kinase activity with a potentially deleterious outcome of the supplements [165]. An age-dependent effect of phytoestrogen supplements is suggested in postmenopausal women [165]. In males, the findings are equivocal and sparse, and more investigations are needed to determine whether the effects will be deleterious or beneficial [165].

## 3. Conclusion

In this article, the efficacy of standardized *Ginkgo biloba* extract, resveratrol, allopregnanolone, and phytoestrogens in combatting age-related cognitive decline has been reviewed. The mechanisms of action as well as preclinical and clinical evidence for each of those entities have been discussed. The four entities share common mechanisms of action but also diverse ones. In terms of the main AD features, A*β* and tau, all four categories were able to reduce the A*β* accumulation but only GBE and phytoestrogens seem to reduce tau hyperphosphorylation. Similarly (and quite predictably due to their phenolic character), all four act as antioxidants either by reducing ROS and oxidative stress (GBE, phytoestrogens, and allopregnanolone) or by enhancing the activity of antioxidant enzymes such as SOD and GPx1 (GBE, resveratrol, and phytoestrogens) and by reducing lipid peroxidation (GBE) and prooxidant enzymes such as Nox4 (resveratrol). GBE, resveratrol, and allopregnanolone target mitochondria by enhancing their functions (activities of complexes, oxidative phosphorylation, oxygen consumption, respiration, mitochondrial membrane potential, and ATP production), while in addition to this, GBE and resveratrol promote mitochondrial biogenesis. This is particularly important since mitochondria play a pivotal role in synaptic plasticity that is reduced in pathological states in the brain. However, there are also some differences in the mechanisms of action of the four discussed substances and mainly in the pathways through which they exert their beneficial effects. Based on our review of the literature, GBE rescues the A*β* neurotoxicity through the activation of the Akt pathway and through phosphorylation of CREB. Neurotrophic factors such as BDNF are stimulated both by GBE and by phytoestrogens. Resveratrol leads to A*β* clearance, enhancement of mitochondrial biogenesis and metabolism, and reduction of inflammation and ROS mainly through the activation of SIRT 1 and AMPK pathways as well as through the deacetylation of PGC-1*α* and the modulation of the JAK/ERK/STAT pathway. Phytoestrogens act as ER receptor modulators. Resveratrol can additionally act as a phytoestrogen and bind to the ER*β* receptor. In terms of *in vitro* assays, it should be taken into account that the extract and the substances should be tested in meaningful, physiologically relevant concentrations and not in irrationally high ones.

Regarding clinical trials, there is a different level of evidence for the four phytochemicals. Standardized GBE, resveratrol, allopregnanolone, and phytoestrogens appear in a descending order according to the level of existing clinical evidence. According to the World Federation of Societies of Biological Psychiatry (WFSBP) Guidelines, GBE has been classified in category B and grade 3 in terms of the outcome of existing studies. Therefore, there is sufficient and good clinical evidence for the efficacy of GBE. There is increasing and promising clinical evidence for resveratrol, but more studies of larger sample size are definitively needed. Lastly, there are no clinical trials indicating the beneficial effect of allopregnanolone and phytoestrogen in age-related cognitive decline disorders. There is only promising evidence from preclinical data regarding allopregnanolone and phytoestrogen. Notably, the four entities follow the same descending order regarding the existing level of clinical evidence and their mitochondria-improving properties. All in all, the effect on mitochondria goes hand in hand with the clinical effect and this highlights one more time the importance of these organelles not only in the pathogenesis of AD but also in aging in general.

## Figures and Tables

**Table 1:** Clinical trials on the effects of GBE.

Study design	GBE dose/preparation	Duration	Subjects	Purpose	Main results	References
Patients with memory complaints, SMI, and MCI						
R, DB, PC	240 mg of GBE once daily or placebo	56 ± 4 days	(61) Healthy aged patients with subjective memory decline (SMI)	Test the effect of GBE on cognitive functions associated with prefrontal dopamine	GBE caused a mild increase in prefrontal dopamine; there were indications for enhanced cognitive flexibility and for ameliorated response inhibition results	Beck et al., 2016 [61]

R, DB, PC	240 mg of GBE once daily or placebo	12 weeks	(300) Patients 45-65 years old with very mild cognitive impairment (MCI)	Evaluate the effects of GBE on cognition and quality of life in patients with very mild cognitive impairment	GBE improved the cognitive ability and quality of life of patients	Grass-Kapanke, 2011 [63]

R, PC, DB, MC	240 mg of GBE once daily or placebo	24 weeks	(160) Patients with MCI	Test the effect of GBE on NPS and cognition in patients with MCI	GBE improved NPS and cognition; the extract was safe and well tolerated	Gavrilova et al., 2014 [62]

Patients with dementia						
R, DB, PC	240 mg of GBE once daily	22 weeks	(400) Demented patients with NPS	Test the efficacy of GBE on NPS of dementia	GBE statistically superior to placebo in ameliorating NPS (e.g., irritability, apathy, and anxiety)	Scripnikov et al., 2007 [64]

Systematic review	240 mg of GBE once daily	22 weeks	(1628) Demented patients with behavioural and psychological symptoms (BPSD)	Demonstrate efficacy of GBE in dementia with BPSD	Improvements of quality of life, cognition, and BPSD activities of daily living clinical global impression	Von Gunten et al., 2016 [66] ([12, 166–168])

Meta-analysis and systematic review	Different dosages of GBE	Not available	Demented patients	Test the efficacy of GBE in ameliorating symptoms of demented patients	GBE improved cognitive function and activities of everyday life in patients with dementia	Brondino et al., 2013 [72] ([12, 67–69, 79, 166, 169, 170])

Meta-analysis of randomized placebo controlled trials	120 mg or 240 mg of GBE per day or placebo	22-26 weeks	(2684) Demented patients	Evaluate evidence for efficacy of GBE in dementia	Confirmation of efficacy of GBE and good tolerability	Gauthier and Schlaefke, 2014 [70] ([12, 69, 166, 167, 169, 171])

Systematic review and meta-analysis of randomized controlled trials	240 mg of GBE once daily	22-26 weeks	(2561) Demented patients	Evaluate the clinical efficacy and adverse effects of GBE in dementia and cognitive decline	GBE was found more effective than placebo in decelerating cognition deficits and in improving daily life activities and NPS in dementia	Tan et al., 2015 [65] ([12, 62, 69, 167, 169–174])

Meta-analysis of randomized controlled clinical trials	240 mg/day	22 or 24 weeks	Old patients aged over 60 years	Effects of GBE on anxiety, dementia, and depression in aging patients	Improvements in dementia, anxiety, and depression	Kasper, 2015 [73] ([12, 166–168])

Meta-analysis of randomized controlled trials	240 mg of GBE once daily	22 or 24 weeks	(1628) Demented patients with behavioural and psychological symptoms (BPSD)	Test the effects of GBE on BPSD of demented patients	Significant superiority of GBE to placebo in improving BPSD and therefore caregiver experience	Savaskan et al., 2017 [74] ([12, 166–168])

Bivariate meta-analysis	Different dosages of GBE	Approximately 6 months	Demented patients	Evaluate baseline risk on the treatment effect and assess the efficacy of GBE on cognitive symptoms of dementia	GBE was effective at improving cognitive functions in dementia after 6 months of treatment	Wang et al., 2010 [71] ([12, 67, 69, 166, 169, 170])

R, DB, PC, PG, MC	160 mg or 240 mg of GBE daily	24 weeks	(214) Patients with dementia or age-related memory loss	To assess the efficacy of GBE in aged demented patients or patients with age-related memory loss	No beneficial effect of GBE for demented or age-related memory-impaired patients	Van Dongen, 2000 [75]

R, DB, PC, PG	120 mg of GBE daily	6 months	176 mildly to moderately demented patients	Assess the efficacy and safety of GBE for treating dementia in early stages	GBE not beneficial in mild to moderate dementia after a 6-month treatment	McCarney et al., 2008 [76]

Cochrane analysis of R, DB, PC trials	Different GBE doses ranging from low to high	Different treatment periods	Aging with dementia or cognitive impairment	Assess the efficacy and safety of GBE in dementia and cognitive impairment	GBE displays unreliable and inconsistent evidence in being beneficial for demented people	Birks and Evans, 2009 [77]

Patients with AD and vascular dementia						
R, DB, PC	120 mg of GBE, 60 mg of GBE, or placebo	6 months	(90) Patients with vascular dementia (VaD)	Evaluate the efficacy and safety of GBE in vascular demented patients	GBE slowed down the cognitive deterioration in vascular demented patients, effect shown in only one of the four neuropsychological tests	Demarin et al., 2017 [78]

Review of R, PC	120 mg of GBE twice daily or 240 mg of GBE once daily	22 or 24 weeks	(1294) Demented patients (AD or VaD) with NPS	Test the efficacy of GBE in older patients with AD/vascular dementia with NPS	Confirmation of efficacy of GBE and good tolerability	Ihl, 2013 [79] ([12, 166, 167, 175])

Systematic review and meta-analysis	GBE extract	12-52 weeks	(2372) Patients with AD or vascular or mixed dementia	Evaluate the effects of GBE in AD and vascular and mixed dementias	Superiority of GBE to placebo in improving everyday life activities in mainly the AD type of dementia	Weinmann et al., 2010 [80] ([67–69, 166, 169, 173, 175])

Systematic review and meta-analysis	240 mg and 120 mg of GBE daily	24 weeks	Patients with MCI or AD	Assess the effectiveness and safety of GBE in treating MCI and AD	There is an indication for the beneficial effect of GBE in MCI and AD but the results were inconsistent	Yang et al., 2016 [81] (AD: [67, 68, 169, 170, 174–176]; MCI: [62])

Systematic review of randomized controlled trials	240 mg of GBE daily	Period ≥ 16 weeks	Patients with mildly to moderately severe and severe AD	Assess the beneficial effect of GBE in AD	Evidence of beneficial effects of GBE in amelioration cognition, every day activities, and psychopathological symptoms but great heterogeneity among the results	Janssen et al., 2010 [82] ([67, 69, 166, 169])

Prevention						
R, DB, PC, PG	120 mg of GBE twice daily	5 years	Adults 70 years or older with occasional memory problems	Efficacy of long-term use of GBE for the prevention of AD in aging with memory complaints	GBE did not reduce the incidence of AD compared to placebo	GuidAge study, Vellas et al., 2012 [83]

R, DB, PC	120 mg of GBE twice daily	Every 6 months from 2000 to 2008	(3069) Healthy old people or people with MCI aged 72 to 96 years	Test whether GBE delays or prevents global or domain-specific cognitive impairment in aging	GBE did not prevent cognitive decline in aging	Snitz et al., 2009 [84]

R, DB, PC	120 mg of GBE twice daily	5 years	(3000) Healthy subjects aging over 80 years old	Assess the ability of GBE in the prevention of dementia in normal aging or those with MCI	GBE does not prevent dementia	GEM study, DeKosky et al., 2006 [87]

Systematic review and meta-analysis	240 mg of GBE daily	Not available	Nondemented patients aged 70 years or older	Evaluate the efficacy of GBE for the prevention of dementia in nondemented adults	GBE is not able to prevent the development of dementia	Charemboon and Jaisin, 2015 [86] ([83, 85])

SMI, subjective memory impairment; MCI, mild cognitive impairments; AD, Alzheimer's disease; VaD, vascular dementia; R, randomized; DB, double blind; PC, placebo controlled; MC, multicenter; PG, parallel group; BPSD, behavioural psychological symptoms; VCI: vascular cognitive impairment. The number of patients involved in the trials is indicated in parentheses.

**Table 2:** Clinical trials on the effects of resveratrol. Ongoing trials are italicized.

Study design	Resveratrol dose/preparation	Duration	Subjects	Purpose	Main results	References
Young and aged healthy individuals						
R, DB, PC, CO	*Trans*-resveratrol from Biotivia Bioceuticals 250 mg or 500 mg	21 days	(24) 18-25 years healthy	Ability to increase cerebral blood flow and modulate mental function	Increase in cerebral flow, no effect in cognitive function	Kennedy et al., 2010 [94]

R, DB, PC, CO	*Trans*-resveratrol 250 mg/day or *trans*-resveratrol 250 mg/day with 20 mg piperine	21 days	(23) Healthy subjects aged 19-34 years	Effect of piperine on the efficacy and bioavailability of resveratrol	Piperine enhances the effect of resveratrol on cerebral blood flow but no effect on bioavailability and cognition	Wightman et al., 2014 [95]

Study in older adults	200 mg of resveratrol per day	26 weeks	(46) Healthy overweight subjects aged 50-75 years	Test whether resveratrol would improve memory performance in older adults	Resveratrol ameliorates memory performance in combination with improved glucose metabolism and increased hippocampal functional connectivity in healthy overweight old people	Witte et al., 2014 [107]

Patients with cognitive decline and postmenopausal women						
R, DB, PC	72 g of active grape formulation	6 months	(10) Adults with mild cognitive decline with mean age of 72.2 years	Evaluate the effects of grapes on regional cerebral metabolism	Grapes could possess a protective effect against early pathologic metabolic decline	Lee et al., 2017 [108]

R, PC, intervention trial	75 mg twice daily of *trans*-resveratrol	14 weeks	(80) Postmenopausal women between 45 and 85 years old	Test the effects of resveratrol on cognition, mood, and cerebrovascular function in postmenopausal women	Resveratrol was well tolerated and able to improve cognition which was related to the improvement of cerebrovascular function. Mood was improved but not significantly.	Evans et al., 2017 [109]

Patients with MCI						
R, DB, interventional study	200 mg of resveratrol per day	26 weeks	(40) Old patients with MCI	Assess if resveratrol improves long-term glucose control, resting-state functional connectivity of the hippocampus, and memory function in patients with MCI	Resveratrol supplementation decreased glycated hemoglobin A1c, preserved hippocampus volume, and improved hippocampus RSFC in patients with MCI	Koebe et al., 2017 [112]

R, DB Phase 1	Bioactive dietary polyphenol preparation (BDPP) at low, moderate, and high doses	4 months	(48) 55-85 years MCI	Safety and efficacy in treating mild cognitive impairment	—	*NCT02502253* [110]

R, DB, PC Phase 4	Resveratrol or omega-3 supplementation or caloric restriction	6 months	(330) 50-80 years MCI	Effects on brain function	—	*NCT01219244* [111]

Patients with mild to moderate AD						
R, DB, PC, MC Phase 2	Resveratrol 500 mg/day with escalation by 500 mg increments ending with 2 g/day	52 weeks	(119) Over 49 years mild to moderate AD	Assess efficacy and safety	No effect on cognitive score, decrease of CSF and plasma A*β*40 levels	Turner et al., 2015 [113]

R, DB, PC Phase 2	Resveratrol 500 mg daily (orally) with a dose elevation by 500 mg every 13 weeks until a final dose of 1000 mg twice daily was reached for the final 13 weeks.	52 weeks	(119) Adults older than 49 years old with a diagnosis of mild to moderate dementia due to AD	Evaluation of safety and tolerability of resveratrol and its effects on AD biomarkers and also on clinical outcomes	Resveratrol was well tolerated and safe, it was detected in the cerebrospinal fluid (nM), it changed the AD biomarker paths, it modified the CNS immune response, and it maintained the BBB integrity; however, more research is needed	*Sawda et al., 2017* [114]

R, DB, PC Phase 3	Longevinex brand resveratrol supplement (resveratrol 250 mg/day)	52 weeks	(50) 50-90 years mild to moderate AD on standard therapy	Effects on cognitive and global functioning	—	NCT00743743 [115]

R, DB, PC Phase 3	Resveratrol with malate and glucose	12 months	(27) 50-90 years mild to moderate AD	Ability to slow the progression of AD	—	NCT00678431 [116]

MCI, mild cognitive impairment; AD, Alzheimer's disease; R, randomized; DB, double blind; PC, placebo controlled; CO, cross over; MC, multicenter; CSF, cerebrospinal fluid. The number of patients involved in the trials is indicated in parentheses.

**Table 3:** Ongoing clinical trial on the effects of allopregnanolone in MCI and mild AD.

Study design	Allopregnanolone dose/preparation	Duration	Subjects	Purpose	Main results	References
R, DB, parallel assignment Phase 1	Allopregnanolone 2, 4, or 6 mg intravenous injection once per week or placebo intravenous injection once per week	12 weeks	(8) For each dose group, 55 years and older, both genders MCI or mild AD (6) Randomized to AP (2) Randomized to placebo	Determine the maximally tolerated dose, safety and tolerability, pharmacokinetic profile, and effects on cognitive function	Not available	NCT02221622 [133]

The number of patients involved in the trials is indicated in parentheses.

**Table 4:** Clinical trials on the effects of phytoestrogens.

Study design	Phytoestrogens dose/preparation	Duration	Subjects	Purpose	Main results	References
Healthy individuals and postmenopausal women						
Randomized control trial	High soya (100 mg total isoflavones/day) or a low soya (0.5 mg total isoflavones/day) diet	10 weeks	(27) Young healthy adults (15 men, 12 women)	Effects on memory, attention, and frontal lobe function	Improvements in short-term memory, long-term memory and mental flexibility	File et al., 2015 [159]
DB, CO, PC	4 capsules/day containing soya isoflavones (116 mg isoflavone equivalents/day: 68 mg daidzein, 12 mg genistein, and 36 mg glycitin) or placebo	6 weeks	(34) Men aged 30-80 years	Effects on cognitive function	Improvements of spatial working memory but no effect on auditory and episodic memory and executive function and visual-spatial processing	Thorp et al., 2009 [160]
18 R, DB, CO, PC	Isoflavone supplementation 60 mg/day or placebo	6 months	(78) Postmenopausal women (mean age 49.5 years)	Effects of soy isoflavones on mood and cognitive function in postmenopausal women	Improvements in mental flexibility, attention, mood, and lower depressive symptoms	Casini et al., 2006 [161]
R, DB, PC	100 mg/day soy isoflavones (glycoside weight) or matching placebo tablets	6 months	Older nondemented men and women (age 62-89 years)	Examination of safety, feasibility, and cognitive efficacy of soy isoflavone administration	Improvements of visual-spatial memory and construction of verbal fluency and speeded dexterity	Gleason et al., 2009 [162]
R, DB, PC	20 g of soy protein containing 160 mg of total isoflavones	12 weeks	(93) Healthy postmenopausal women (mean age 56 years)	Effect of a high-dose isoflavones on cognition, quality of life, lipoproteins, and androgen status in postmenopausal women	Significant improvement in the quality of life versus placebo. No significant effects in cognition. The testosterone and HDL levels were significantly lower at the end of the study.	Basaria et al., 2009 [163]

The number of patients involved in the trials is indicated in parentheses.

## Abbreviations

17*β*-estradiol: Estradiol
2A PP2Ac: Catalytic subunit of protein phosphatase
3xTgAD: Triple transgenic mouse model of AD
AD: Alzheimer's disease
Akt: Protein kinase B
AMPK: 5′ adenosine monophosphate-activated protein kinase
APP: Amyloid precursor protein
ATP: Adenosine triphosphate
A*β*: beta-Amyloid protein
*α*KGDH: *α*-Ketoglutarate dehydrogenase
Bax: Apoptotic regulator
BBB: Blood-brain barrier
Bcl-2: Anti-B-cell lymphoma 2
BDNF: Brain-derived neurotrophic factor
BDPP: Bioactive dietary preparation
Ca^2+^: Calcium
CMRglc: Cerebral metabolic rates of glucose
COX-2: Cyclooxygenase-2
CREB: Cyclic AMP response element-binding protein
CSF: Cerebrospinal fluid
CTRL: Untreated SH-SY5Y cells
DAT: Dopamine transporters
E2: Estrogen
ER: Endoplasmic reticulum
ERT: Estrogen replacement therapies
ETC: Electron transport chain
FAD: Familial Alzheimer's disease
FOXO: Forkhead box O
GBE: *Gingko biloba* extract
GPx1: Glutathione peroxidase 1
GSK3*β*: Glycogen synthase kinase 3 beta
hNSCs: Human neural stem cells
HO-1: Heme oxigenase 1
*I*_*A*_: Transient potassium channel
IBO: Ibotenic acid
IKK: Inhibitory kappa B kinase
IMM: Inner mitochondrial membrane
IMR-32: Human neuroblastoma cells
iNOS: Nitric oxide synthase
JAK/ERK/STAT: Janus kinases/extracellular signal-regulated kinases/signal transducers and activators of transcription
JNK: c-Jun N-terminal kinase
LXR: Liver-X-receptor
MCI: Mild cognitive impairment
MDA: Malondialdehyde
MMP: Mitochondrial membrane potential
MnSOD: Manganese superoxide dismutase
mPTP: Mitochondrial permeability transition pore
MRI: Magnetic resonance imaging
MTDLs: Multitarget-directed ligands
mtDNA: Mitochondrial DNA
NF-*κ*B: Nuclear factor kappa-light-chain-enhancer of activated B cells
Nox4: Nicotinamide adenine dinucleotide phosphate (NADPH) oxidase 4
NPS: Neuropsychiatric symptoms
NRF-2: Nuclear factor erythroid 2-related factor 2
NRs: Nuclear receptors
OVX: Ovarectomized
OXPHOS: Oxidative phosphorylation
p53: Tumor protein
P450scc: Cytochrome cholesterol side-chain cleavage enzyme
PAF-AH-1: Platelet-activating factor-acetylhydrolase-1
PCG-1*α*: Peroxisome proliferator-activated receptor *γ* coactivator-1*α*
PC12: Pheochromocytoma cells
PDH: Pyruvate dehydrogenase
PKC-*δ*: Protein kinase C delta
PPAR*γ*: Peroxisome proliferator-activated receptor gamma
PSD95: Synapse-associated protein
PXR: Pregnane xenobiotic receptor
RNS: Reactive nitrogen species
ROS: Reactive oxygen species
RSFC: Resting-state functional connectivity
SAD: Sporadic Alzheimer's disease
SAMP1: Senescence-accelerated mice
SGZ: Subgranular zone
SH-SY5Y: Human neuroblastoma cells
SIRT1: Sirtuin1
SMI: Subjective memory impairment
SOD: Superoxide dismutase
SOD1: Superoxide dismutase 1
TH: Tyrosine hydroxylase
TNF-*α*: Tumor necrosis factor alpha
TrkA: Tropomyosin receptor kinase A
TrkB: Tropomyosin receptor kinase B
TSPO: The translocator protein
TyrRS-PARP1: Tyrosyl tRNA synthetase-poly(ADP-ribose) polymerase 1
VaD: Vascular dementia
YY-1224: A terpene trilactone-enhanced GBE.
